# Loss of AF-6/afadin induces cell invasion, suppresses the formation of glandular structures and might be a predictive marker of resistance to chemotherapy in endometrial cancer

**DOI:** 10.1186/s12885-015-1286-x

**Published:** 2015-04-12

**Authors:** Takuro Yamamoto, Taisuke Mori, Morio Sawada, Hiroshi Matsushima, Fumitake Ito, Makoto Akiyama, Jo Kitawaki

**Affiliations:** Department of Obstetrics and Gynecology, Kyoto Prefectural University of Medicine, 465 Kajii-cho, Kawaramachi Hirokoji, Kamigyo-ku, Kyoto 602-8566 Japan

**Keywords:** AF-6/afadin, Endometrial cancer, Invasion, Morphology, ERK, Src

## Abstract

**Background:**

AF-6/afadin plays an important role in the formation of adherence junctions. In breast and colon cancer, loss of AF-6/afadin induces cell migration and cell invasion. We aimed to elucidate the role of AF-6/afadin in human endometrial cancer.

**Methods:**

Morphology and AF-6/afadin expression in endometrial cancer cell lines was investigated by 3-dimensional culture. We used Matrigel invasion assay to demonstrate AF-6/afadin knockdown induced invasive capability. Cell proliferation assay was performed to estimate chemoresistance to doxorubicin, paclitaxel and cisplatin induced by AF-6/afadin knockdown. The associations between AF-6/afadin expression and clinicopathological status were determined by immunohistochemical analysis in endometrial cancer tissues. Informed consent was obtained from all patients before the study.

**Results:**

The majority of cell clumps in 3-dimensional cultures of Ishikawa cells that strongly expressed AF-6/afadin showed round gland-like structures. In contrast, the cell clumps in 3-dimensional cultures of HEC1A and AN3CA cells—both weakly expressing AF-6/afadin—showed irregular gland-like structures and disorganized colonies with no gland-like structures, respectively. AF-6/afadin knockdown resulted in reduced number of gland-like structures in 3-dimensional cultures and enhancement of cell invasion and phosphorylation of ERK1/2 and Src in the highly AF-6/afadin-expressing endometrial cancer cell line. Inhibitors of MAPK/ERK kinase (MEK) (U0126) and Src (SU6656) suppressed the AF-6/afadin knockdown-induced invasive capability. AF-6/afadin knockdown induced chemoresistance to doxorubicin, paclitaxel and cisplatin in Ishikawa cells, not in HEC1A. Immunohistochemical analysis showed that AF-6/afadin expression was significantly associated with myometrial invasion and high histological grade.

**Conclusions:**

AF-6/afadin regulates cell morphology and invasiveness. Invasive capability is partly regulated through the ERK and Src pathway. The inhibitors to these pathways might be molecular-targeted drugs which suppress myometrial invasion in endometrial cancer. AF-6/afadin could be a useful selection marker for fertility-sparing therapy for patients with atypical hyperplasia or grade 1 endometrioid adenocarcinoma with no myometrial invasion. AF-6/afadin knockdown induced chemoresistance especially to cisplatin. Therefore, loss of AF-6/afadin might be a predictive marker of chemoresistance to cisplatin.

## Background

Endometrial cancer is one of the most common gynecological malignancies and its incidence has increased remarkably [1,2]. Endometrial cancers are broadly classified into two groups: (1) Type 1 disease, the most common type of endometrial cancer, is estrogen-related, low-grade, histologically endometrioid adenocarcinoma in most cases and shows minimal myometrial invasion and occurs at a younger age. (2) Type 2 disease is high-grade, histologically serous or clear cell adenocarcinoma and shows deep myometrial invasion [3].

Cell polarity and cell-cell adhesion are essential for normal functioning of epithelial tissues. In cancer, the epithelial-mesenchymal transition (EMT) is a process where epithelial cells detach from primary tumors, invade into the surrounding tissues, metastasize, and grow at a secondary site [4]. Cell-cell junctions are lost in EMT. Most patients present with low-grade and early-stage endometrial cancer. However, once the disease spreads beyond the uterus, the prognosis is poor, and the 5-year survival is 25–45% for stage III and IV [5]. Therefore, it is essential to decrease tumor invasiveness for the treatment of endometrial cancer.

AF-6/afadin is encoded by the MLLT4 gene located on chromosome 6, band q27 [6]. AF-6/afadin binds to nectins, plays important cooperative roles in the formation of adherens junctions, and is associated with the actin cytoskeleton [7]. It is also important for cell polarity at cell-cell junctions [8,9]. Conversely, AF-6/afadin at the leading edge does not bind nectins, and enhances cell movement [10]. Thus, AF-6/afadin has conflicting role in cell invasion. AF-6/afadin loss induces cell migration, invasion, and proliferation, and is a prognostic indicator in breast and colon cancer [11-14]. However, its role and expression in endometrial cancer have not been studied. In this study, we investigate for the first time the expression of AF-6/afadin in patients with endometrial cancer and its role in cell invasion and chemoresistance in endometrial cancer.

## Methods

### Cell lines and materials

The Ishikawa line of human uterine endometrial cancer cells was provided by the Cell Resource Center for Biomedical Research (Institute of Development, Aging and Cancer, Tohoku University, Japan). The HEC1A and AN3CA cells were purchased from the American Type Culture Collection. The Ishikawa and AN3CA cells were maintained in Eagle’s MEM (Nacalai Tesque, Kyoto, Japan) with nonessential amino acids, sodium pyruvate, and 10% fetal bovine serum (FBS) (Invitrogen Corp., Carlsbad, CA). The HEC1A cells were maintained in McCoy’s 5A (HyClone, Logan, UT) with sodium pyruvate, supplemented with 10% FBS. We focused on endometrioid adenocarcinoma because it was the major type observed, and the histological grade and myometrial invasion are important for its diagnosis and treatment. All patient samples used for this study were obtained from University hospital, Kyoto Prefectural University. The Kyoto Prefectural University of Medicine human research ethics board approved all protocols and patients gave informed consent.

### RNA isolation and quantitative PCR analysis

Total RNA (1 μg) was isolated from the cells, 24 or 48 h after siRNA transfection, using the RNeasy mini kit (Qiagen, Hilden, Germany) according to the manufacturer’s instructions. Each cDNA was synthesized from 1 μg RNA using the ReverTra Ace qPCR RT kit (Toyobo, Osaka, Japan). Real-time reverse transcription-PCR was carried out using the CFX Connect™ Real-Time System (Bio-Rad, Hercules, CA). cDNA samples prepared from the total RNA of Ishikawa, HEC1A, and AN3CA cells (1 μL) were mixed in 20-μL reactions containing SYBR qPCR Thunderbird master mix (Toyobo, Osaka, Japan) and 0.2 μmol/L of each primer. Primers, AF-6/afadin 5′-GTGGGACAGCATTACCGACA-3′ (forward) and 5′-TCATCGGCTTCACCATTCC-3′ (reverse) and Glyceraldehyde 3-phosphate dehydrogenase (GAPDH) 5′-GCACCGTCAAGGCTGAGAAC-3′ (forward) and 5′-ATGGTGGTGAAGACGCCAGT-3′ (reverse) were designed with Primer 3 software. The amplification, detection, and data analysis were performed using the CFX Connect™ Real-Time System. Each sample was analyzed in triplicate. The expression levels of genes were determined relative to the expression level of GAPDH.

### RNA interference

Small interfering RNAs (siRNA) for MLLT4/AF-6 (s8829, s8830 and s8831) and a negative control siRNA (control #1) targeting no known genes were Silencer® Select siRNAs purchased from Ambion (Austin, TX). Cells were transfected with the siRNA using Lipofectamine RNAiMAX (Invitrogen, Carlsbad, CA) according to the manufacturer’s instructions, and used for each experiment after 24 or 48 h. AF-6/afadin KD effects were measured with real-time PCR and western blotting. We decided to use siRNA for AF-6/afadin (s8830) for the following experiments, because it has the strongest KD activity of the validated siRNAs.

### Three-dimensional cell culture

Cells (2.5 × 10^5^) from the Ishikawa, HEC1A, and AN3CA lines were trypsinized and suspended in 800 μL of BD Matrigel™ Matrix Basement Membrane (BD biosciences, Bedford, MA), as previously described [15]. The paraffin-embedded specimens were cut into 3.5-μm sections for hematoxylin-eosin staining and AF-6/afadin immunohistochemical staining. Each experiment was performed three times.

### Matrigel invasion assay

Cells (2.0 × 10^5^) were seeded into the top of a Matrigel invasion chamber (24-well insert; pore size, 8 μm; BD Biosciences, Bedford, MA) containing a serum-free and a medium with 10% FBS was used as a chemoattractant in the lower chamber. The cells were incubated for 48 h at 37°C. A cotton swab was used to remove the cells, that did not invade through the pore and cells that had migrated to the lower surface of the membrane were stained with the Diff-Quik kit (Sysmex, Kobe, Japan) and counted.

### Cell proliferation assay

Cells (5.0 × 10^3^) were seeded into 96-well plates containing a normal growth medium, and RNA interference was performed after 24 h. The anticancer drugs were added at various doses, 24 h after siRNA transfection. The cells were cultured and treated in quadruplicate, and cell viability was examined after 72 h by the 2-(2-methoxy-4-nitrophenyl)-3-(4-nitrophenyl)-5-(2,4-disulphonyl)-2H-tetrazolium (WST-8) assay (Nacalai Tesque, Kyoto, Japan).

### Antibodies

A mouse anti-AF-6/afadin antibody (clone 35) was purchased from Becton Dickinson. A mouse anti-Src antibody (# 2110) and rabbit anti-GAPDH (# 2118), anti-ERK1/2 (# 9102), anti-phospho-ERK1/2 (Thr202/Tyr204), and anti-phospho-Src family (Tyr416) (# 2101) antibodies were purchased from Cell Signaling Technology (Beverly, MA). All antibodies were used at the concentration recommended by the manufacturers.

### Western blotting

Cells were washed twice in phosphate-buffered saline and lysed in RIPA buffer (Nacalai Tesque, Kyoto, Japan).

Cell lysates (20 μg) were heated in sodium dodecyl sulfate (SDS) sample buffer (125 mM Tris–HCl, pH 6.8, 4% SDS, 25% glycerol, 10% 2-mercaptoethanol, 0.05 mM phenylmethanesulfonyl fluoride and 0.004% bromophenol blue), separated using 10% e-PAGEL according to the manufacturer’s recommendations (Atto Corp, Tokyo, Japan), and transferred onto Immuno-Blot® PVDF membranes (Bio-Rad, Hercules, CA). The membranes were blocked in Tris-buffered saline supplemented with 5% fat-free milk for 1 h and then incubated with indicated antibodies at 4°C overnight. After washing, the membranes were incubated with the secondary antibody for 1 h at room temperature. The signal was developed using Chemi-Lumi One Super (Nacalai Tesque, Kyoto, Japan) and analyzed by a ChemiDoc XRS system with Image Lab software (Bio-Rad, Hercules, CA).

### Immunohistochemistry

Specimens from patients who underwent abdominal hysterectomy because of uterine endometrial cancer were used for this study. Informed consent was obtained from all the patients before the study was conducted. The protocol has been previously described [16]. The AF-6/afadin immunoreactivities were scored using a semiquantitative index, the H-score. The H-score is the product of the intensity of staining (given a value of 0, 1, 2, or 3 for negative, weak, moderate, or strong, respectively) and the percentage of stained epithelial cells at each intensity (0–100%). Samples with H-score ≥ 50 were deemed as AF-6/afadin-positive.

### Statistical analysis

Progression-free survival and overall survival were assessed using the Kaplan-Meier method and log-rank test. Comparisons of the means and standard error of data between two groups were performed using the Student’s *t* test. Comparisons of over 3 groups were performed using the Kruskal-Wallis H-test, and the Mann–Whitney U-test with Bonfferoni correction was as a post hoc test. Statistical differences between AF-6/afadin status and histological type, histological grade, myometrial invasion, lymph node status, and stage were evaluated using a chi-square test. P values < 0.05 were considered significant.

## Results

### Low expression levels of AF-6/afadin in uterine endometrial cancer tissues were associated with myometrial invasion and high histological grade

All sections of normal endometrium stained positive for AF-6/afadin. AF-6/afadin was expressed in the cytoplasm and at the surface of the epithelial cells. The association between AF-6/afadin immunoreactivity and clinicopathological status are shown in Table 1. Of the 90 endometrial cancer cases, 85 cases were endometrioid adenocarcinomas.

**Table 1:** **Association between AF-6/afadin immunoreactivity and clinicopathological parameters in 90 uterine endometrial cancers**

AF-6/afadin immunoreactivity			
Uterine endometrial cancer	Positive (n = 51)	Negative (n = 39)	*P*value
Mean age (range)	55.9 (32–83)	58.6 (28–80)	0.41
Histological type			
Endometrioid	51 (100%)	34 (87.2%)	
Serous	0	3 (7.7%)	
Clear	0	2 (5.1%)	0.21
Endometrioid adenocarcinoma	Positive (n = 51)	Negative (n = 34)	*P* value
Histological grade			
1	38 (74.5%)	11 (32.4%)	
2	11(21.6%)	13(38.2%)	
3	2 (3.9%)	10 (29.4%)	*P* < 0.01
Myometrial invasion			
No invasion	14 (27.5%)	1 (2.9%)	
Less than half	26 (51.0%)	15 (44.1%)	
More than half	11 (21.6%)	18 (52.9%)	*P* < 0.01
Lymph node metastasis			
Negative	48 (94.1%)	30 (88.2%)	
Positive	3 (5.9%)	4 (11.8%)	0.57
Stage			
I	37 (72.5%)	25 (73.5%)	
II	7 (13.7.8%)	2 (5.9%)	
III	6 (11.8%)	6 (17.6%)	
IV	1 (2.0%)	1 (2.9%)	0.82

Cases with H-score > 50 are positive.

**Figure 1:**
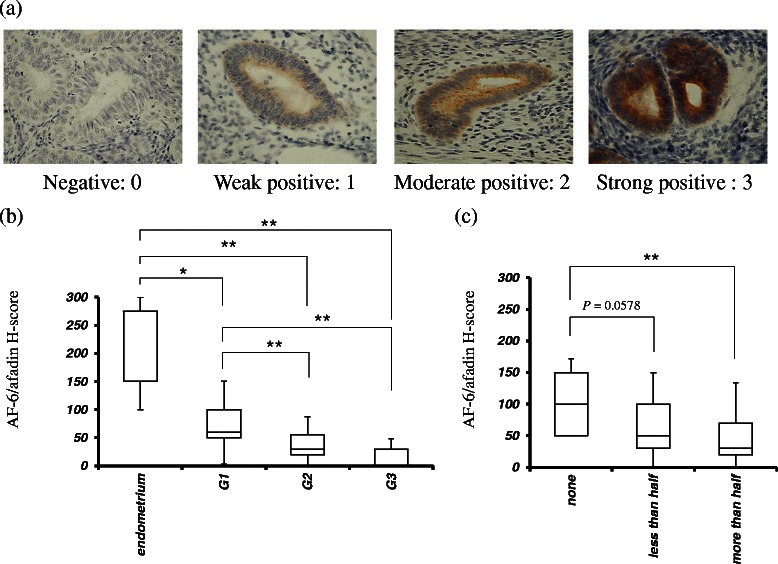
Association of AF-6/afadin expression with histological grade and myometrial invasion in 85 endometrioid adenocarcinomas. **(a)** The staining values of 0, 1, 2, or 3 are negative, weak, moderate, or strong, respectively. **(b, c)** Low H-score of AF-6/afadin immunoreactivity was associated with high histological grade (between grade 1 and 2 cases, and between grade 1 and 3 cases, *P* < 0.01, and 0.01, respectively) and deep myometrial invasion (between no invasion and more than half invasion cases, *P* < 0.01) **(b)**. The correlations between AF-6/afadin H-score and histological grade or myometrial invasion were assessed with the Kruskal-Wallis H-test; the Mann–Whitney *U*-test with Bonfferoni correction was used as a post hoc test. Significant differences were indicated as * for *P* < 0.05 and ** for *P* < 0.01.

The H-score of AF-6/afadin was significantly associated with histological grade (between grade 1 and 2 cases, and between grade 1 and 3 cases, *P* < 0.01) and myometrial invasion (between cases with no invasion and cases with a myometrial invasion depth of more than 50%, *P* < 0.01) in endometrioid adenocarcinoma (Figure 1b, c). The H-score of the normal endometrium (n =10) was significantly higher than that of the endometrioid adenocarcinoma (between normal endometrium and grade 1, *P* < 0.05, and between normal endometrium and grade 2, and 3, *P* < 0.01) (Figure 1b). However, there were no significant associations between the AF-6/afadin immunoreactivity and the FIGO stage, lymph node metastasis, progression-free survival (PFS), or overall survival (OS) (*P* = 0.18 and 0.21 for PFS and OS, respectively).

### AF-6/afadin plays an important role in 3-dimensional cultures of Ishikawa, HEC1A, and AN3CA cells

**Figure 2:**
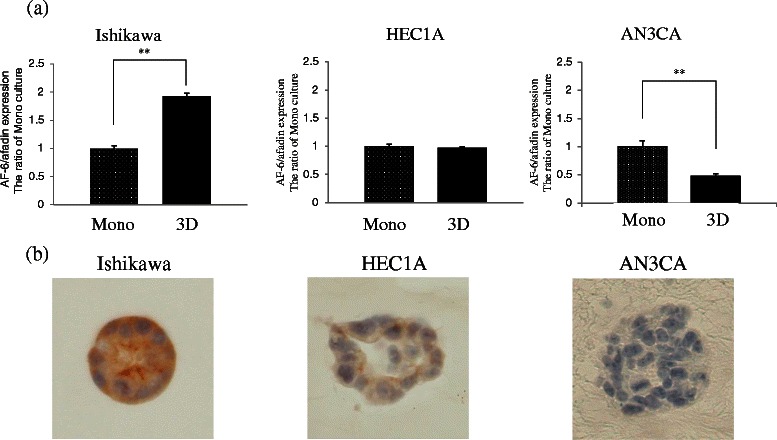
Three-dimensional (3D) cultures of Ishikawa, HEC1A, and AN3CA cells. **(a)** Real-time RT-PCR for AF-6/afadin using cDNA from Ishikawa, HEC1A, and AN3CA cells, standardized with GAPDH. AF-6/afadin mRNA levels in 3D culture were significantly higher than that in monolayer (Mono) culture in the Ishikawa cells, remained unchanged from that in Mono culture in the HEC1A cells, and was significantly lower than that in Mono culture in the AN3CA cells. Significant difference were indicated as ** for *P* < 0.01. **(b)** AF-6/afadin immunohistochemistry of 3D culture showed that Ishikawa, HEC1A, and AN3CA cells were strongly positive, weakly positive and very weakly positive, respectively. A large portion of the cell clumps formed round gland-like structures in 3D cultures of Ishikawa cells, whereas in 3D cultures of HEC1A and AN3CA cells, irregular gland-like structures and in the form of disorganized colonies with no gland-like structures, respectively, were observed.

**Figure 3:**
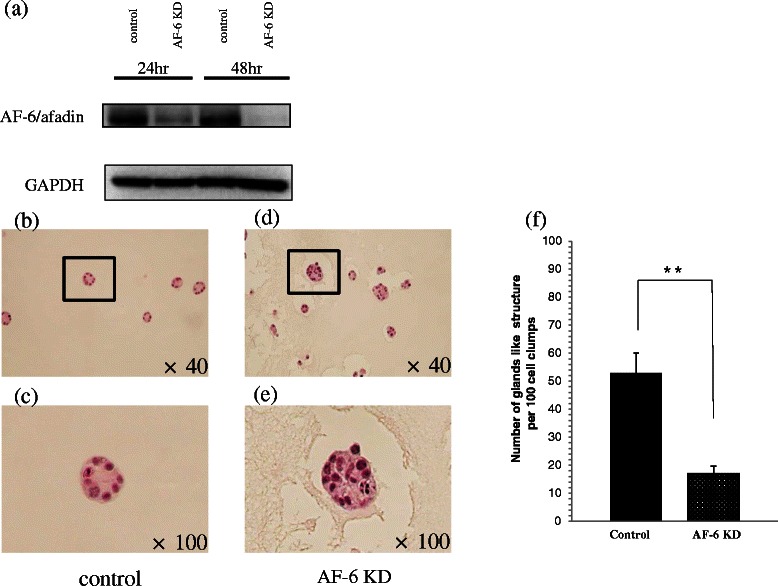
Morphology change induced by AF-6/afadin KD in the Ishikawa cells in 3D culture. The paraffin-embedded samples were cut into 3.5-μm sections. Immunohistochemistry was performed to confirm the efficacy of AF-6/afadin KD (data not shown) and **(a)** we also examined AF-6/afadin KD efficacy by western blotting. **(b, c)** The morphological differences in 3D cultures were assessed by hematoxylin-eosin staining. The KD control Ishikawa cells mainly formed the glands like structures. Panel **(c)** is the magnification of the box in **(b)**. **(d, e)** AF-6/afadin KD Ishikawa cells mainly formed disorganized colonies. Panel **(e)** is magnification of the box in **(d)**. **(f)** The number of gland-like structures per 100 cell clumps was counted three times. AF-6/afadin KD significantly reduced the number of gland-like structures. Significant differences were indicated as ** for *P* < 0.01.

To investigate AF-6/afadin expression and morphology, 3-dimensional (3D) culture was performed. AF-6/afadin mRNA level in 3D cultures was significantly higher than that in monolayer cultures in Ishikawa cells (*P* < 0.01), similar to that in monolayer cultures in the HEC1A, and significantly lower than that in monolayer cultures in AN3CA cells (*P* < 0.01) (Figure 2a). AF-6/afadin immunostaining was strongly, weakly, and very weakly positive in the Ishikawa, HEC1A, and AN3CA cells, respectively. The majority of the cell clumps in 3D cultures of Ishikawa cells showed round gland-like structures, whereas the cell clumps in 3D cultures of HEC1A and AN3CA showed irregular gland-like structures and disorganized colonies with no gland-like structures, respectively (Figure 2b). RNA interference experiments to knockdown (KD) AF-6/afadin and subsequent 3D cultures were performed using the Ishikawa cells. In this assay, paraffin specimens were prepared on day 5 when the AF-6/afadin KD was still maintained. AF-6/afadin KD increased the number of disorganized colonies and reduced the number of gland-like structures in 3D cultures of Ishikawa cells (*P* < 0.01) (Figure 3).

### AF-6/afadin KD induced phosphorylation of ERK1/2 and Src kinase and stimulated cell invasion in AF-6/afadin-positive cell lines

**Figure 4:**
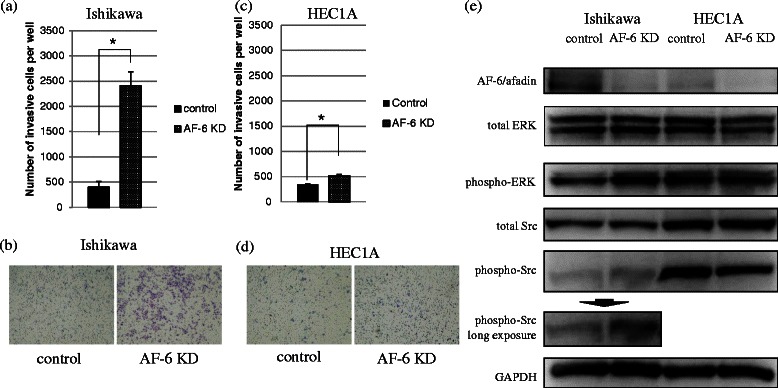
Invasiveness of Ishikawa cells is enhanced by AF-6/afadin KD through phosphorylation of ERK1/2 and Src. Invasiveness was evaluated using the Matrigel invasion assay. For this assay, siRNA for control or AF-6/afadin was transfected into the Ishikawa or HEC1A cells. **(a-d)** The number of invasive cells increased significantly in AF-6/afadin KD Ishikawa and HEC1A cells. Significant differences are indicated as * for *P* < 0.05. **(e)** Expression of GAPDH was used as the internal control. Using siRNA-negative control or siRNA against AF-6/afadin, AF-6/afadin was effectively knocked down at the protein level. AF-6/afadin KD induced phosphorylation of ERK1/2 and Src in the Ishikawa cells, whereas expression of phosphorylated ERK1/2 and Src was already strong and was not up-regulated by AF-6/afadin KD in HEC 1A cells.

First, we evaluated whether AF-6/afadin regulates cell migration and invasion in endometrial cancer, using trans-well cell culture inserts and the Matrigel Invasion chamber system. We used Ishikawa (AF-6/afadin strong positive) cells and HEC1A (AF-6/afadin weak positive) cells. AF-6/afadin KD cells significantly enhanced the invasive capability in the Ishikawa and HEC1A compared with negative control cells (*P* < 0.05) (Figure 4a-d); however, although the migratory capability was examined using Trans-well cell culture inserts without Matrigel coating, it remained unchanged (data not shown).

**Figure 5:**
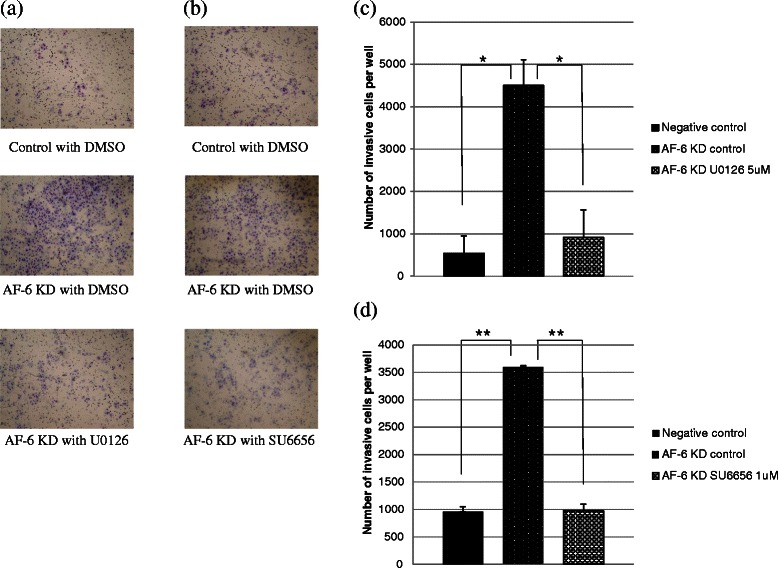
Effect of U0126 and SU6656 on invasiveness induced by AF-6/afadin KD in the Ishikawa cells. **(a, c)** AF-6/afadin KD enhanced cell invasion and U0126 inhibited the invasive capability of Ishikawa cells. **(b, d)** SU6566 also showed the inhibitory effect.

We next investigated how AF-6/afadin KD stimulates invasive capability. In Ishikawa cells, AF-6/afadin KD induced phosphorylation of ERK1/2 and Src kinases. Conversely, in HEC1A cells, ERK1/2 and Src kinase were already activated and AF-6/afadin KD did not significantly increase the level of phosphorylated ERK1/2 or Src (Figure 4e). This result indicates that AF-6/afadin functions as a suppressor of ERK and Src phosphorylation pathways. Using the MEK inhibitor (U0126) and the Src kinase inhibitor (SU6656), we demonstrated the importance of ERK1/2 and Src phosphorylation for regulating the cell invasion induced in the Ishikawa cells by AF-6/afadin KD. First, we determined the concentrations of U0126 and SU6656 (5 μM and 1 μM, respectively). At the determined concentrations, the reagents inhibited phosphorylation of ERK1/2 and Src, and did not influence the proliferation of the Ishikawa cells (data not shown). At these concentrations, U0126 and SU6656 significantly inhibited Ishikawa cell invasions induced by AF-6/afadin KD (Figure 5). This result indicates that AF-6/afadin KD induced cell invasion through ERK and Src signaling pathways.

### AF-6/afadin KD induced chemoresistance to doxorubicin, paclitaxel and cisplatin in AF-6/afadin strongly positive cell line

**Figure 6:**
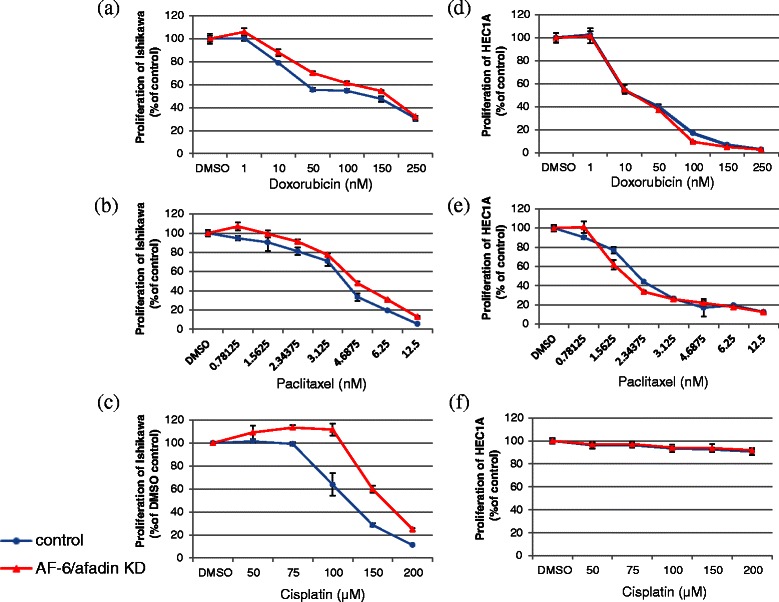
AF-6/afadin KD-induced chemoresistance to doxorubicin, paclitaxel, and cisplatin induced. Blue lines show proliferation of cells transfected with control siRNA, and red lines show AF-6/afadin KD. **(a-c)** AF-6/afadin KD induced slight chemoresistance to doxorubicin and paclitaxel, and strong chemoresistance to cisplatin in Ishikawa cells **(d-f)**; however, it did not induce chemoresistance in HEC1A cells.

To estimate chemoresistance to doxorubicin, paclitaxel and cisplatin induced by AF-6/afadin KD, we performed the WST-8 assay cell proliferation assay using. In Ishikawa cells, AF-6/afadin KD induced slight chemoresistance to doxorubicin and paclitaxel, and strong resistance to cisplatin (Figure 6a-c, respectively). However, AF-6/afadin did not induce chemoresistance in HEC1A cells (Figure 6d-e).

## Discussion

AF-6/afadin is expressed in almost all normal epithelial tissues where it is involved in forming the actin cytoskeleton by binding actin filaments and nectins (immunoglobulin-like cell adhesion molecules) [17]. AF-6/afadin, in cooperation with nectins, involved in the formation of a variety E-cadherin-dependent or E-cadherin-independent cell-cell junctions [7,9,18,19]. Decreased E-cadherin expression is associated with poor prognosis, high histological grade and advanced stage in endometrial cancer [20-22]. Immunohistochemical analysis revealed that AF-6/afadin expression in endometrial cancer tissues was lower than that in normal endometrial tissues. Furthermore, loss of AF-6/afadin expression was associated with myometrial invasion and high histological grade in patients with endometrial cancer.

Three-dimensional cell culture is a useful model to investigate molecular signaling and cellular behavior during epithelial morphogenesis [23]. In this study, when comparing the mRNA levels in monolayer and 3D cultures, the mRNA levels of nectins in both cultures were found to be similar, while AF-6/afadin mRNA levels in well-differentiated and poorly differentiated endometrial cancer cell lines were higher and lower, respectively, in the 3D cultures. These results suggest that AF-6/afadin could be an important scaffold protein determining epithelial morphogenesis in uterine endometrial cancer.

AF-6/afadin was strongly expressed in Ishikawa cells, a well-differentiated and grade 1 equivalent endometrial adenocarcinoma cell line, whereas AF6/afadin was weakly expressed in HEC1A and AN3CA cells, which are derived from grade 2 and grade 3 endometrial adenocarcinomas, respectively. In 3D cultures, AF-6/afadin expression was positively associated with the formation of round gland-like structures. AF-6/afadin was expressed in all normal endometrial tissues and most well-differentiated endometrial adenocarcinomas in our study. Therefore, we investigated the function of AF-6/afadin using an RNA interference assay. In endometrioid cancers, histological grade is defined in terms of solid tumor growth; increased solid growth is associated with high histological grade and a high degree of cellular atypia [24]. AF-6/afadin KD resulted in fewer gland-like structures and more disorganized colonies in Ishikawa cell cultures than in the KD control cultures. This result explains why AF-6/afadin loss was associated with higher histological grade. AF-6/afadin KD significantly enhanced cell invasion in AF-6/afadin-positive endometrial cancer cell lines. These results suggest that AF-6/afadin serves as a positive regulator of duct formation and as an inhibitor of tissue invasion in human endometrial cancer.

We focused on the cell signaling pathways induced by AF-6/afadin KD to determine the mechanisms underlying these findings. A Src signaling is important in multiple physiological homeostatic pathways that regulate cell proliferation, cell survival, cytoskeleton regulation, intracellular contacts, cell-matrix adhesion, motility, and migration [25,26]. An Src mutation at codon 537 was previously detected in a small subset of endometrial cancers [27]. In this study, we demonstrated that enhancement of phosphorylation in the Src kinase family was induced by AF-6/afadin KD in the strongly AF-6/afadin-positive Ishikawa cells. The AF-6/afadin KD-induced invasiveness of the Ishikawa cells was repressed by SU6656, an Src inhibitor. These findings indicate that AF-6/afadin might regulate cell invasion through the Src signaling pathway.

RAS/RAF/MEK/ERK pathways are also important for cell proliferation and survival in several cancers [28-33]. AF-6/afadin links to Bcr and RAS and down-regulates RAS-dependent stimulation of the RAF/MEK/ERK signaling pathway [34]. The activation of ERK1/2 induced by AF-6/afadin KD has been reported in breast cancer [12]. We also found that AF-6/afadin KD increased phosphorylation of ERK1/2 proteins. In addition, U0126, a MEK inhibitor, also suppressed AF-6/afadin KD induced phosphorylation of ERK1/2 and cell invasion in endometrial cancer. These results also suggest that AF-6/afadin KD induced cell invasion through the RAF/MEK/ERK signaling pathway.

Chemoresistance is an important problem in cancer therapy. The ERK pathway has been implicated in chemoresistance to doxorubicin, paclitaxel and cisplatin in some cancers [35-39], although the findings are controversial. AF-6/afadin KD increased the phosphorylation of ERK 1/2 in Ishikawa cells, but not in HEC1A cells. AF-6/afadin KD also induced chemoresistance to doxorubicin, paclitaxel, and cisplatin in AF-6/afadin strongly positive Ishikawa cells. These results suggest that the ERK pathway could be involved in the chemoresistance induced by AF-6/afadin KD.

## Conclusions

In this study, we demonstrated that AF-6/afadin regulates cell invasion through ERK and Src pathway. The inhibitors to these pathways might be molecular-targeted drugs which suppress myometrial invasion in endometrial cancer. AF-6/afadin is an important scaffold protein and might be crucial for maintenance of the ductal structure of glands in the endometrium. Reduced expression of AF-6/afadin is associated with high histological grade and myometrial invasion of endometrial cancers. AF-6/afadin could be a useful selection marker for fertility-sparing therapy for the patients with atypical hyperplasia or grade 1 endometrioid adenocarcinoma with no myometrial invasion. AF-6/afadin KD induced strong chemoresistance to cisplatin. Therefore, loss of AF-6/afadin might be a predictive marker of chemoresistance to cisplatin.

## Abbreviations

3D: Three-dimensional
EMT: Epithelial-mesenchymal transition
KD: Knockdown
PFS: Progression-free survival
OS: Overall survival
FBS: Fetal bovine serum
siRNA: Short interfering RNA
